# Inter-Observer Agreement in Measuring Respiratory Rate

**DOI:** 10.1371/journal.pone.0129493

**Published:** 2015-06-19

**Authors:** Louise Gramstrup Nielsen, Lars Folkestad, Jacob Broder Brodersen, Mikkel Brabrand

**Affiliations:** 1 Department of Anaesthesiology, Sygehus Lillebaelt, Vejle, Denmark; 2 Department of Endocrinology, Odense Universitetshospital, Odense, Denmark; 3 Department of Medicine, Sydvestjysk Sygehus, Esbjerg, Denmark; 4 Department of Emergency Medicine, Sydvestjysk Sygehus, Esbjerg, Denmark; New York University School of Medicine, UNITED STATES

## Abstract

**Background:**

Respiratory rate (RR) is an important vital sign which is strongly correlated with in-hospital mortality. At the same time, RR is the most likely vital sign to be omitted when assessing a patient. We believe that one reason for this could be the difficulty in measure the RR, since it is not read off a monitor, but counted manually. Also there is the possibility of assessment bias and the inter-observer reliability becomes important. We therefore set out to investigate how the nursing staff counting the actual number of respirations per minute would agree with the nursing staff using a predefined ordinal scale.

**Methods:**

For this prospective study, we recorded five videos of a young healthy man breathing approximately 5, 10, 15, 30 and 60 times per minute. The videos were shown in a random order to a suitable sample of the nursing staff. The participants were randomized into two groups; one to count the exact number of breaths per minute, and one to use a predefined ordinal scale.

**Results:**

Comparing the exact number of breaths per minute, the Intra Class Coefficient (ICC) was 0.99 (95% CI: 0.97–1.00). Comparing the RR using the predefined scale, the overall Kappa Fleiss Coefficient was 0.75.

**Conclusions:**

The inter-observer agreement was high when comparing the use of the actual number of breaths per minute and substantial when comparing the use of the predefined scale. This is the largest inter-observer study on RR to date. However, further studies on the use of scaled comparisons of RR are needed.

## Introduction

Respiratory rate (RR) is an important vital sign in the assessment of patients and has proven to be strongly correlated with in-hospital mortality and serious adverse events (e.g. cardiac-pulmonary arrest or ICU admissions) [1, 2]. Some authors even argue that RR is a stronger predictor of mortality than blood pressure or pulse [3, 4]. However, as RR is most often manually counted as an exact number of breaths per minute, the results are error prone.

Also, several studies have illustrated that RR measurements are omitted when vital signs are collected at a daily basis during admission in acute hospital wards [1], medical- and surgical wards [5] and general medical admission units [6].

Some argue that a reason for not measuring the RR is a lack of knowledge of its importance. It has been suggested that nurses and doctors should be educated better to appreciate the RR as an useful marker in assessing patients in risk of serious adverse events [1]. Others suggest that RR is not measured because there is no automated respiratory measuring device outside the intensive care unit, and instead the pulse oximetry is used in assessing the respiratory state of patients [7].

It therefore also becomes challenging and time consuming to measure RR, making the measurement more laborious and therefor tempting to omit.

One method to increase the reporting of RR might be to introduce an ordinal-scaled measurement of the RR as very slow, slow, normal, fast and very fast.

The majority of vital signs are measured electronically, which reduces the risk of assessment bias. However, when a measurement in clinical medicine is not captured electronically, the risk of assessment bias (e.g. miscalculations or perhaps misunderstood methodology) is suddenly present. It therefore becomes important to assess the reliability of the measurement. Previous studies into the inter-observer reliability of RR measurements are based on few observers assessing the patients [7–10]. Since RR in reality can change rapidly, and it is not possible to have a long queue of observers standing in line to assess several patients, previous studies have asked the observers to assess one patient simultaneously or within a short time frame. Using the videos, we have the possibility of asking many observers to assess the same patient simultaneously, thus limiting the assessment bias.

With the present study, we aimed at investigating the inter-observer agreement between nursing staff measuring the RR by counting the exact number of breaths per minutes or by using a predefined ordinal scale. We hypothesized that the use of a predefined ordinal scale would result in a higher inter-observer agreement compared to the group counting the exact number of breaths.

## Methods

We examined the inter-observer agreement in measuring the RR. And as most vital signs, including RR, are assessed by the nursing staff, the observers in our study were nurses and nurses assistants at a medical admission unit in Denmark.

**Fig 1:**
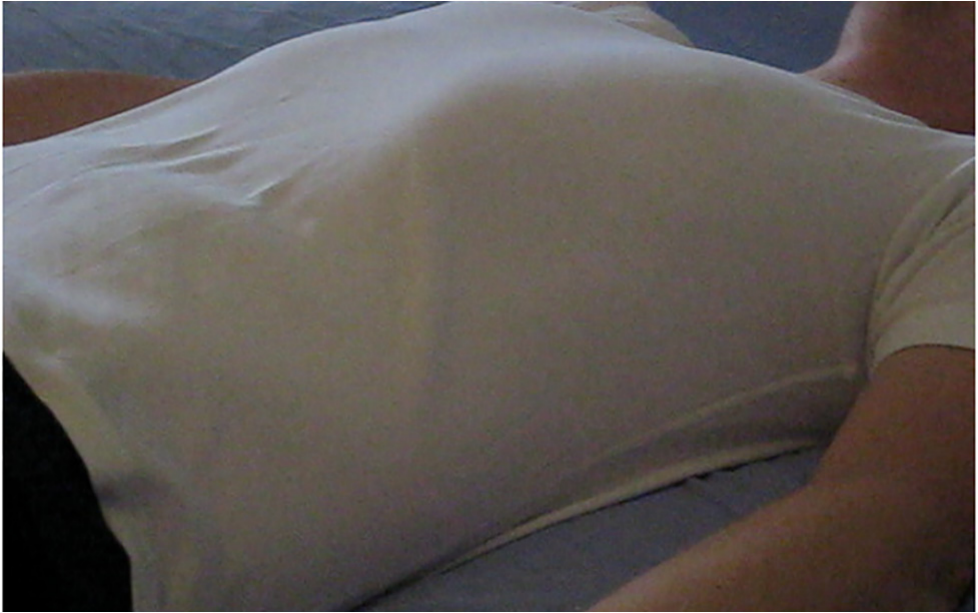
Picture from one of the videos, showing the volunteer lying down, wearing a white t-shirt on a dark background.

The study is a questionnaire study based on video recordings of one patient (simulated). We recorded five videos using a Canon IXUS 70 camera (Canon Danmark A/S, Søborg Denmark). The videos showed a healthy thirty years old male breathing respectively 5, 10, 15, 30 and 60 times per minute. A picture from the videos can be seen in Fig 1. To ensure that the simulated patient did indeed take the assigned number of breaths, several practice runs were conducted with the camera operator counting the number of breaths out loud. The simulated patient was supine and wearing a white t-shirt on a dark background. Only the thorax and neck was shown. Each video lasted approximately 70 seconds. The videos were shown to the participants at a teaching session for nurses and nurses assistants working at the emergency department or medical admissions unit at the hospital Sydvestjysk Sygehus, Esbjerg, Denmark. Each participant was unable to see the other participants answers, and each saw all five videos. When the video was playing the participants could hear the simulated patients breaths. This method has previously been described in detail regarding capillary refill time, proving this method efficient [11].

The videos were shown to a convenient sample of participants consisting of nurses and nurses assistants. The participants were randomized into two groups by handing out alternate forms for them to complete. 18 were asked to count the exact number of breaths per minute, and 19 to use the predefined scale of very slow, slow, normal, fast and very fast. When using the ordinal scale, the participants were not given any indication of where a given RR should be allocated on the scale.

### Statistics

The data is presented as median (range). The Intra Class Coefficient (ICC) was used to classify the agreement between the participants measuring the exact numbers of breaths per minute. Fleiss Kappa statistics were used to assess the reproducibility (reliability) of the measurements when comparing the RR in the group using the predefined ordinal scale. Kappa values greater than 0.75 represents an excellent agreement. Kappa values between 0.4–0.75 represents a fair to good agreement, and values less than 0.4 a moderate to poor agreement. There are no established criteria for sample size calculations for this type of study, and therefore none were performed.

Finally we stratified the participants into two groups according to experience (above and below the median experience). The agreement between the experienced and the less-experienced was tested by using a Wilcoxon-Rank-Sum test for the counted RR. For the scaled RR we used Spearman’s chi-squared test. A p-value > 0.05 indicates no significant difference in the RR noted by the two groups. In other words, the two groups agree on the RR noted, if the p-value is above 0.05.

A statistical analysis was performed using Stata version 11 (Stata Corp., Texas, USA).

### Ethical considerations

According to Danish law, an Institutional Review Board (IRB) (Videnskabsetisk komite Region Syddanmark) approval was not required. The IRB was contacted prior to the study and a waiver of approval was given. The participants were not offered any incentive to participate in the study. All participants gave oral informed consent that their responses were recorded for scientific purposes. Due to the waiver of approval from the IRB, no written consent was obtained. All participants were informed that participation was voluntary; all asked participants wanted to participate. We kept no records of participant consent, since a filled out questionnaire would indicate informed consent to participate.

## Results

Four nurses assistants and 34 nurses participated in the study. The median age of the participants was 42 (25–63) years, and the median experience was 18 (0–36) years.

Overall, the results showed high agreement between the observers.

For the group of observers counting the exact RR, the Intraclass Coeffiecient was calculated to 0.99 (95% CI: 0.97–1.00). For video 1 (real RR 60) the participants counted the RR to be a median of 64 breaths per minute (50–70), for video 2 (real RR 10) 8 (8–10), for video 3 (real RR 5) 5 (4–7), for video 4 (real RR 15) 10 (9–11) and for video 5 (real RR 30) 31 (30–32). When using Wilcoxon-Rank-Sum there were no significant differences between the experienced and less-experienced groups in the counted RR for each video.

In the group using our predefined ordinal-scale, the overall Fleiss Kappa was calculated to be 0.75, which also indicates a high inter-observer agreement. Kappa was calculated for video 1 at 0.94, video 2 at 0.55, video 3 at 0.78, video 4 at 0.61 and video 5 at 0.88. For video 2, 80% of the participants reported the RR to be normal, 15% for it to be slow, and 5% reported the RR to be very slow. For video 4, 75% of the participants reported the RR to be normal, 20% for it to be slow, and the last 5% reported the RR to be very slow. The lowest Kappa was calculated for video 2 and 4, indicating a fair and substantial inter-observer agreement. Using Spearman’s chi-squared test all p-values were above 0.05.

**Fig 2:**
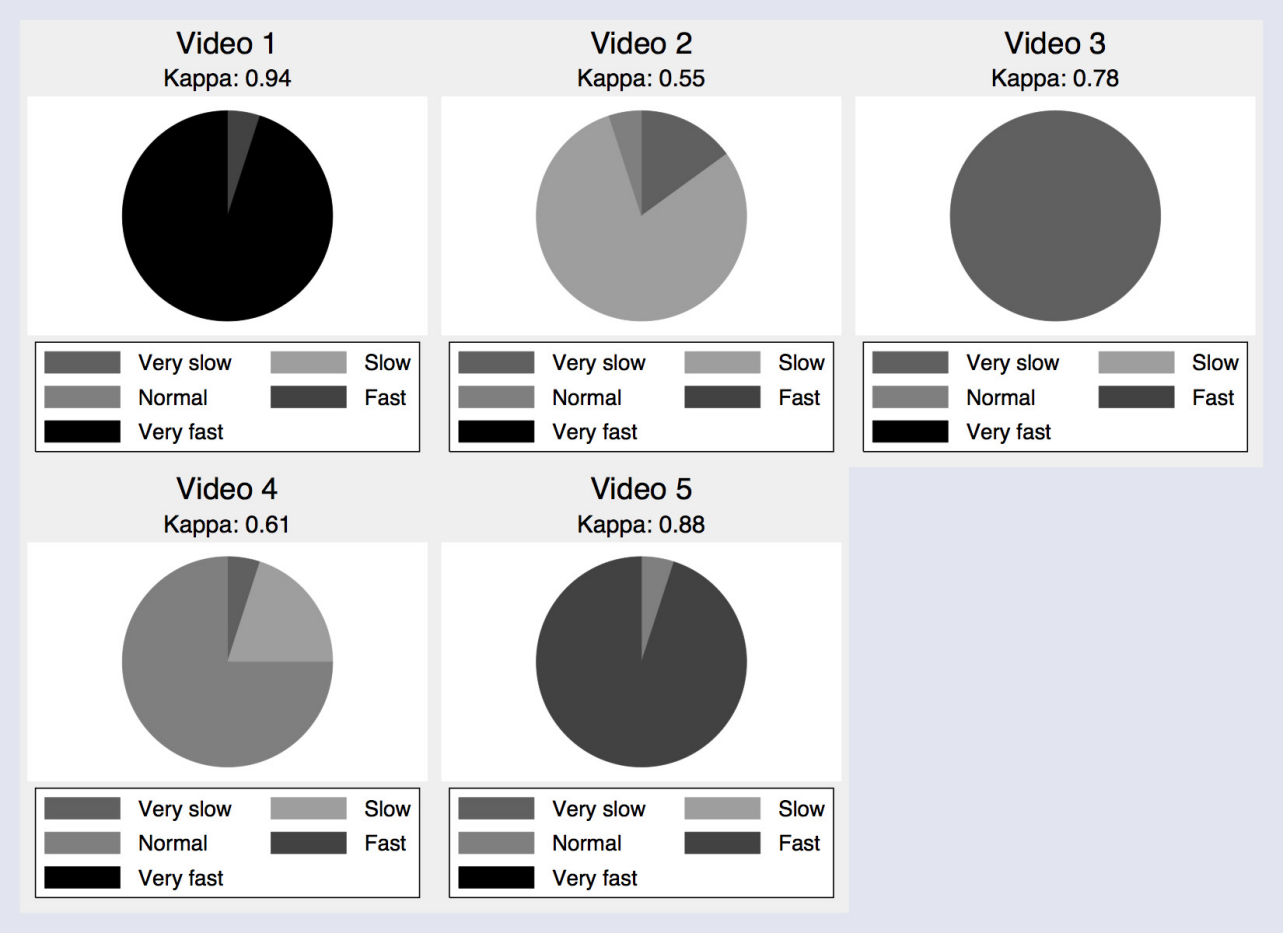
Pie chart of RR reported on an ordinal scale, showed for each video.

**Fig 3:**
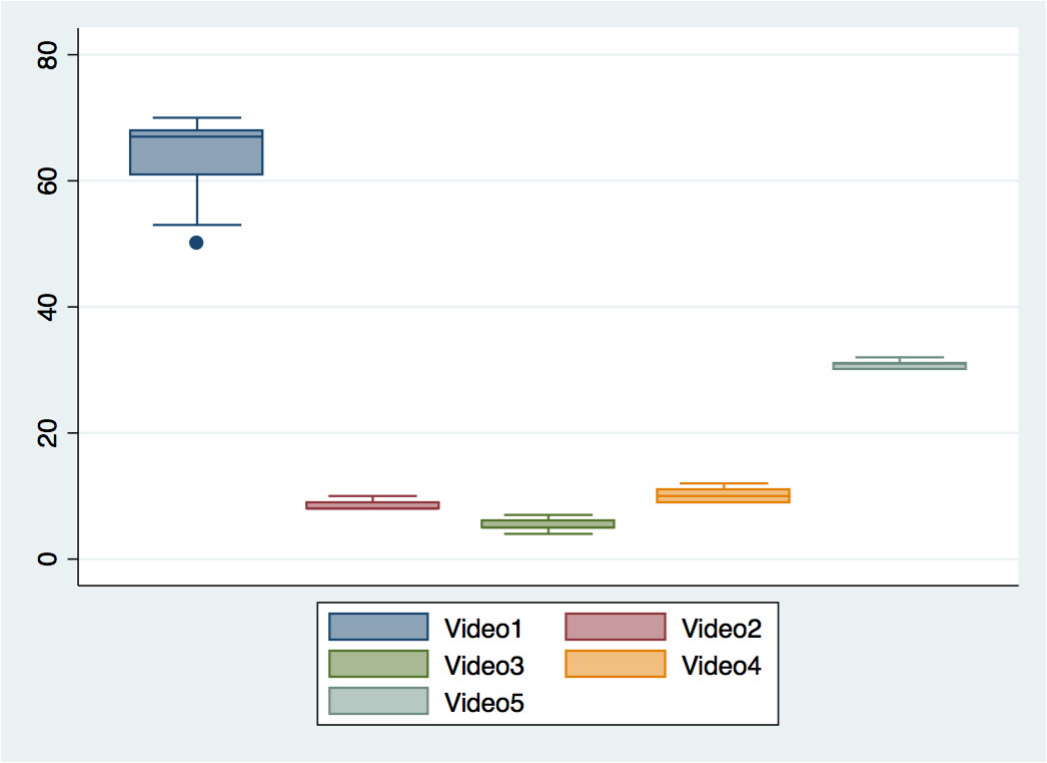
Boxplot indicating the median and range of RR when reported as an exact rate, showed for each video.

Fig 2 shows the results reported from using the ordinal scale, and Fig 3 shows a box-plot of the exact counted RR. Table 1 shows the nurses and nurses assistants noted RR for each video, and whether the participant were counting the RR or using the scale.

**Table 1:** Results.

Nurse/Nurses assistant RR-Counted	Nurse/Nurses assistant RR-Scaled	Video 1 RR Counted (RR = 60)	Video 1 RR Scaled	Video 2 RR Counted (RR = 10)	Video 2 RR Scaled	Video 3 RR Counted (RR = 5)	Video 3 RR Scaled	Video 4 RR Counted (RR = 15)	Video 4 RR Scaled	Video 5 RR Counted (RR = 30)	Video 5 RR Scaled
Nurse	Nurses assistant	50	Fast	10	Slow	6	Very Slow	12	Slow	30	Normal
Nurse	Nurse	53	Very Fast	8	Very Slow	5	Very Slow	11	Normal	30	Fast
Nurse	Nurse	60	Very Fast	8	Slow	5	Very Slow	10	Slow	30	Fast
Nurse	Nurse	61	Very Fast	8	Slow	5	Very Slow	9	Normal	30	Fast
Nurse	Nurse	61	Very Fast	8	Slow	5	Very Slow	10	Normal	30	Fast
Nurses assistant	Nurse	62	Very Fast	9	Slow	5	Very Slow	10	Normal	31	Fast
Nurse	Nurse	66	Very Fast	8	Very Slow	5	Very Slow	10	Very Slow	31	Fast
Nurses assistant	Nurse	67	Very Fast	8	Slow	5	Very Slow	9	Normal	30	Fast
Nurse	Nurse	67	Very Fast	8	Slow	5	Very Slow	9	Normal	31	Fast
Nurse	Nurse	67	Very Fast	8	Normal	6	Very Slow	10	Slow	31	Fast
Nurse	Nurse	67	Very Fast	9	Slow	6	Very Slow	11	Normal	31	Fast
Nurse	Nurse	68	Very Fast	9	Slow	5	Very Slow	11	Normal	32	Fast
Nurse	Nurse	68	Very Fast	8	Slow	6	Very Slow	11	Normal	31	Fast
Nurse	Nurse	68	Very Fast	10	Slow	4	Very Slow	10	Normal	31	Fast
Nurse	Nurse	68	Very Fast	8	Slow	6	Very Slow	11	Normal	31	Fast
Nurses assistant	Nurse	68	Very Fast	8	Very Slow	7	Very Slow	10	Normal	31	Fast
Nurse	Nurse	68	Very Fast	8	Slow	5	Very Slow	9	Normal	31	Fast
Nurse	Nurse	70	Very Fast	8	Slow	5	Very Slow	9	Normal	30	Fast
.	Nurse	.	Very Fast	.	Slow	.	Very Slow	.	Slow	.	Fast
.	Nurse	.	Very Fast	.	Slow	.	Very Slow	.	Normal	.	Fast

. no participant

## Discussion

We investigated the inter-observer agreement by having one simulated patient and multiple observers. When asking 38 nurses and nurses assistants to assess the RR, we found a high inter-observer agreement in the group counting the exact RR. Furthermore, we found substantial inter-observer agreement in the group using our predefined ordinal scale. Overall this gives the impression of a high inter-observer agreement in assessing the RR.

We expected the inter-observer agreement to be the highest in the group using the predefined scale. This was not confirmed by our results, however. It is noteworthy that the lowest Kappa values are calculated in video 2 and 4. Video 2 shows the simulated patient breathing approximately 10 times per minute, and video 4 shows a RR of 15 breaths per minute. The explanation for the lowest kappa values calculated in video 2 and 4, we believe, is due to the small difference in the RR when comparing the scenarios in those to videos. When having to assess the RR without actually counting the exact numbers of breaths per minute, the small difference becomes harder to detect. Since a RR of 10 and 15 is close to what is considered the normal range of the RR, it is even more difficult for the observers to differentiate between the two categories (slow and normal). For video 1, the simulated patient had a RR of 60, and one nurses assistant noted the RR to be fast, not very fast. For video 5, one nurse noted the RR to be normal even though the real RR was 30. For video 4, one nurse noted the RR to be very slow even though the real RR was 15. If the RR is estimated wrongly, too high or too low, it could lead to under- or over- estimating the patients illness, and can then lead to an incorrect triage, monitoring and treatment. The participants were given no guidance to which levels of RR were to be categorized as very-slow to very-fast. This could account for some of the misclassifications of the RR. Experience in patient treatment and observation could also lead to some differences in the classification of the RR. However, we found no difference between the most experienced and least experienced participants regardless of using the counted- or scaled RR.

Overall, our results confirm earlier findings. In a study by Lim et al, the RR was calculated in 245 patients in medical wards or attending the lung function department. Each patient was assessed twice with 15 minutes between the assessments. They found a very good agreement between the observers in the RR measurements [8]. In another study by Worster et al, the inter-observer agreement was also good with no significant difference in the RR between the observers. The patients were patients admitted to the emergency department at an urban hospital in central Canada, and the RR assessment was a part of the standard triage at admission. The first assessment of the RR was made by the triage nurse, and the second assessment was made 2 minutes later by a clinical physician [12]. Liu et al however found the lowest inter-observer agreement in measuring the RR with Kappa values between 0.36–0.53 in a study testing a clinical score (including RR, retractions, dyspnoea and auscultations), assessing the respiratory status in children hospitalized with asthma and bronchiolitis [9]. Why Liu et al found a lower degree of agreement between the observers compared to other studies, is unknown. One reason for this could be that the children were crying, making it more difficult to count the exact RR.

Our study has limitations. The simulated patient recorded in the videos was breathing with a fixed RR during each video sequence, and it was therefore not true to reality, making our study a simulation more than a clinical study. In reality, the RR changes quickly according to the clinical condition, and is thus difficult to compare between observers, unless counted simultaneously. Another limitation is the fact that the participants were fully aware of the purpose of the video sequences, and therefore may have been more thorough in the assessment than they would during clinical practice. In addition, there is a risk of incomplete blinding, and the videos were subject to lighting, blurring and colours.

The main strength of our study is that the observer is the only variable, as multiple observers assess the same patient simultaneously. At the same time, we eliminate the possible natural variation and uncertainty of few observers performing independent observations consecutively by using video recordings. Removing the bias of changing the conditions between the observations provides a more accurate impression of the actual inter-observer agreement than designs used in previous studies.

## Conclusion

We found high inter-observer agreement when assessing the exact RR, and substantial agreement when using a predefined ordinal scale. We expected the inter-observer agreement to be higher when using the predefined scale, but this was not confirmed in our study. Our predefined scale, however, needs further analysis before possible implementation in standard clinical use. Whether an ordinal scale will increase the recording of the RR is unknown, but the study shows that nurses and nurses assistants agree on how to record and report the RR.
